# When Are New Hippocampal Neurons, Born in the Adult Brain, Integrated into the Network That Processes Spatial Information?

**DOI:** 10.1371/journal.pone.0017689

**Published:** 2011-03-09

**Authors:** C. Jimena Sandoval, Marisela Martínez-Claros, Paola C. Bello-Medina, Oswaldo Pérez, Víctor Ramírez-Amaya

**Affiliations:** 1 Laboratorio de redes neuronales plásticas, Departamento de Neurobiología Conductual y Cognitiva, Instituto de Neurobiología, Universidad Nacional Autónoma de México, Campus Juriquilla, Querétaro Qro, México; 2 Laboratorio de neurofisiología de la percepción, Departamento de Neurobiología Conductual y Cognitiva, Instituto de Neurobiología, Universidad Nacional Autónoma de México, Campus Juriquilla, Querétaro Qro, México; Mental Health Research Institute and the University of Melbourne, Australia

## Abstract

Adult-born neurons in the dentate gyrus (DG) functionally integrate into the behaviorally relevant hippocampal networks, showing a specific Arc-expression response to spatial exploration when mature. However, it is not clear when, during the 4- to 6-week interval that is critical for survival and maturation of these neurons, this specific response develops. Therefore, we characterized Arc expression after spatial exploration or cage control conditions in adult-born neurons from rats that were injected with BrdU on one day and were sacrificed 1, 7, 15, 30, and 45 days post-BrdU injection (PBI). Triple immunostaining for NeuN, Arc, and BrdU was analyzed through the different DG layers. Arc protein expression in BrdU-positive cells was observed from day 1 to day 15 PBI but was not related to behavioral stimulation. The specific Arc-expression response to spatial exploration was observed from day 30 and 45 in about 5% of the BrdU-positive cell population. Most of the BrdU-positive neurons expressing Arc in response to spatial exploration (∼90%) were located in DG layer 1, and no Arc expression was observed in cells located in the subgranular zone (SGZ). Using the current data and that obtained previously, we propose a mathematical model suggesting that new neurons are unlikely to respond to exploration by expressing Arc after they are 301 days old, and also that in a 7-month-old rat the majority (60%) of the neurons that respond to exploration must have been born during adulthood; thus, suggesting that adult neurogenesis in the DG is highly relevant for spatial information processing.

## Introduction

New neurons born in the adult mammal Dentate Gyrus (DG) functionally integrate into the hippocampal network [1], [2], [3]. The integration process resembles the one described during early development [4], [5] but is slower [6], [7]. During the first 4 weeks ∼80% of the new neurons die, and the remaining 20% survive for at least 11 months [8]. Thus, the first 4 weeks are critical for the survival of these new neurons and also for their maturation [9], [10]. For example, in the first week after birth new neurons are partially differentiated [11] and express doublecortin (DCX), which is important for neuronal migration [12], and by the third week ∼90% of the new neurons express NeuN, a marker of mature neurons [12], [8]. New neurons do not show electrophysiological features of maturity until the third week [13], [14]: their GABAergic response, which is initially depolarizing, becomes hyperpolarizing around this time [15], [16], and the glutamatergic input matures during weeks 3 and 4 [13], [14], [15]. Anatomically, the axon, dendrites, and their spines reach maturity around weeks 3 to 4 [17], [14], [18].

By detecting the expression of the immediate early gene (IEG) Arc induced by spatial exploration, we previously examined the functional integration of 5-month-old, adult-born granular neurons into the behaviorally relevant hippocampal network [2]. We found that more adult-born neurons expressed Arc in animals allowed to explore, than in cage control animals, indicating that adult-born neurons have a specific Arc-expression response to spatial exploration [2]. Nevertheless, it is not clear when this specific response to exploration appears.

By detecting the expression of the IEGs cFos and Arc evoked during a water-maze task in previously trained animals, Kee and colleagues [19] observed that 6-week-old new neurons, but not younger ones, are recruited into circuits that can be re-activated at 10 weeks. They also showed that the expression of cFos after a single water-maze session occurs only in 6-week- but not in 1-week-old new neurons, similar to previous findings showing cFos expression after seizures only in 3-week-old new neurons [20]; this earlier work was the first to show responsiveness of adult-born granular neurons to spatial behavior [20]. Additionally, Tashiro and colleagues found that when they are 2 weeks old, new neurons are preferentially recruited into circuits that process information from experience in an enriched environment, which enhances new neuron survival [21].

However, the question remains as to when, during this critical period for survival and maturation, do adult-born neurons integrate into the behaviorally relevant hippocampal network, developing a specific response to spatial exploration? The expression of the IEG Arc and its protein product, stimulated by behaviorally induced neural activity [22] and important for synaptic plasticity [23], [24], is detected as early as 24 hours after the birth of adult-born granular neurons [25]. Therefore, this question can be answered by comparing Arc protein expression in new granular neurons 30 minutes after spatial exploration or cage control conditions during the critical time for survival and maturation, without affecting these processes by behavioral stimulation.

**Figure 1:**
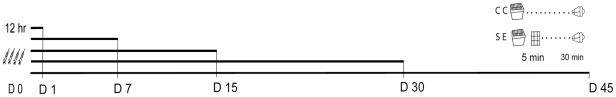
Schematic representation of the experimental procedure. BrdU was administered on day 0, in 4 separate ip injections (50 mg/Kg each) every 4 h from 9:00 A.M to 9:00 P.M. Animals were sacrificed 1, 7, 15, 30, or 45 days post BrdU injections (PBI), either from their home cages (cage control, CC) or 30 min after a 5-min spatial exploration (SE).

**Figure 2:**
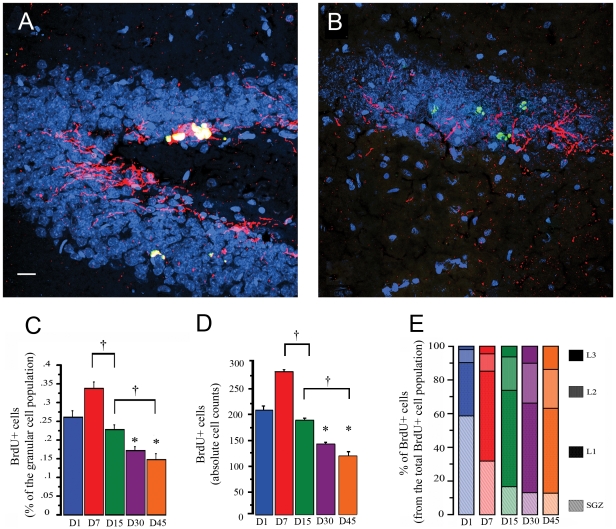
Survival and migration of adult-born granular cells. Confocal images, taken with a 25× objective, of the DG stained with DCX in red, BrdU in green, and counterstained with DAPI in blue. A) shows BrdU-positive cells co-localizing (yellow) with DCX from an animal sacrificed 7 days PBI; B) shows BrdU-positive cells from an animal sacrificed 45 days PBI that do not co-localize with DCX. C) Average proportion of BrdU-positive cells from the total granular cell population found in each (1, 7, 15, 30, and 45 days) PBI group. *P<0.01 as compared to day 1 PBI group, † P<0.01 between indicated groups. Note the significant decrease in the proportion of BrdU-positive cells found with time after new neurons were born. D) Absolute BrdU-positive cell counts found in each PBI group (1, 7, 15, 30 and 45 days); note that the results are very similar to the proportions shown in C. E) Percentage of BrdU-positive cells from the total BrdU-positive cell population found in each DG layer: the subgranular zone (SGZ), DG layer 1 (L1), DG layer 2 (L2), and DG layer 3 (L3), for the different PBI times. The different proportions of BrdU-positive cells found in the various layers with increasing time suggest that new cells migrate through the DG layers.

**Figure 3:**
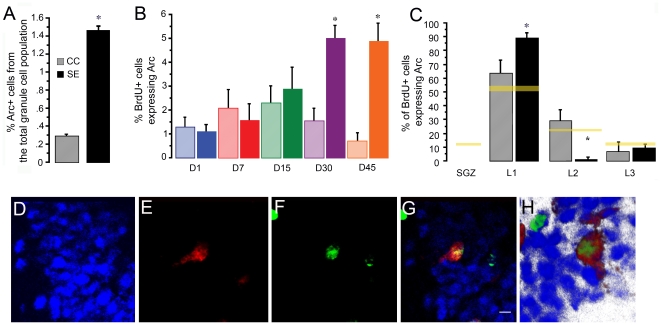
Arc expression after spatial exploration. A) Percentage of the whole granular cell population that expresses Arc after spatial exploration. Note that Arc expression is observed in a higher proportion of cells in the exploration group (solid bar) than in cage controls (pattern bar). *P<0.001. B) Percentage of BrdU-positive cells that expressed Arc in each PBI group, i.e., sacrificed either directly from its home cage (CC, pattern bar) or after spatial exploration (SE, solid bar). Arc expression in BrdU-positive cells occurs with no behavioral stimulation from day 1 until day 15 PBI, but on days 30 and 45 PBI, Arc expression was observed in significantly more BrdU-positive neurons from exploration animals than from cage controls. *P<0.01. C) Percentage of BrdU-positive cells expressing Arc in each DG layer, either after cage control conditions (patterned bar) or spatial exploration (solid bar). The yellow horizontal bars represent the proportion of the total BrdU-positive cells found in each layer on days 30 and 45. Note that no Arc expression was found in BrdU-positive neurons in the SGZ. In L1, the IEG Arc was expressed in a larger proportion of new neurons from exploration animals than from the cage controls, *P<0.01, indicating that BrdU-positive neurons located in L1 are more likely to respond to exploration. The opposite was observed in L2, i.e. the proportion of BrdU-positive cells in L2 that expressed Arc was much lower in SE than in CC animals. D to H) 40× confocal images taken from the middle plane of a confocal microscope image stack were used to verify the co-localization of Arc and BrdU. D) NeuN in blue, E) Arc protein in red, F) BrdU in green, G) NeuN, Arc, and BrdU merge; note that BrdU-positive neurons expressing Arc appear yellow. H) Flat image obtained from a 3D boxels reconstruction. The rotation of the 3D projection was used to confirm BrdU and Arc co-localization. The scale bar in G is 100 μm.

**Figure 4:**
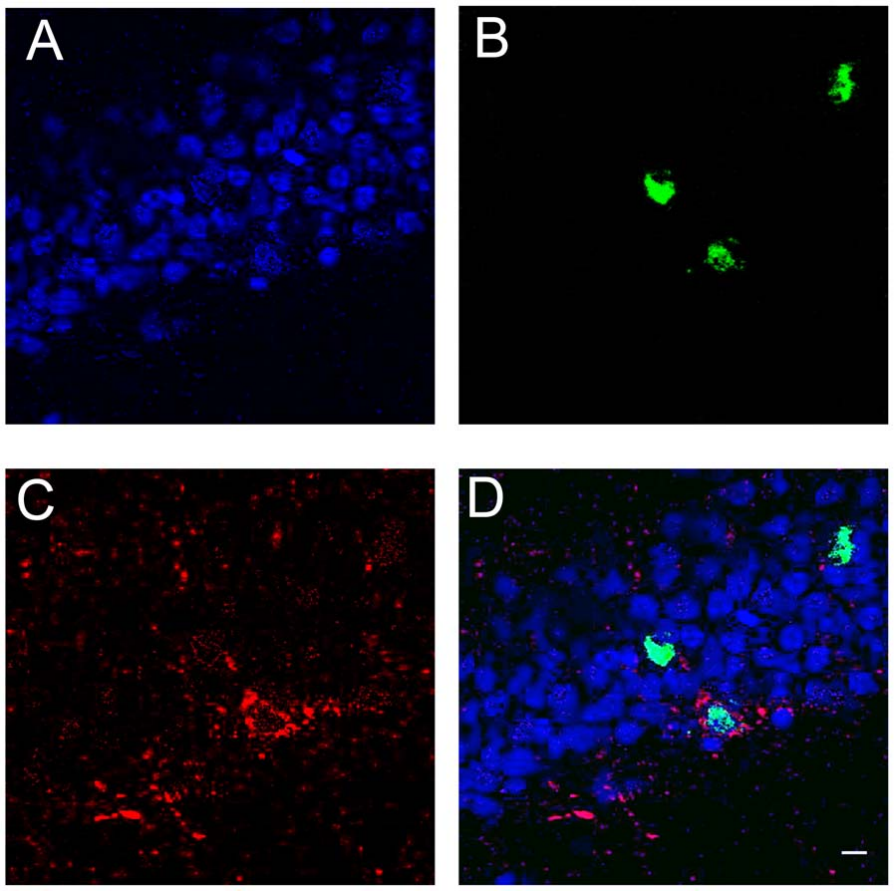
Arc expression in DCX-positive cells. The images show double immunohistochemistry for Arc and DCX, and were taken with the Apotome microscope system (Zeiss) with a 40×/1.3 NA objective using Z section resolution. A) DAPI is shown in blue, B) Arc protein is shown in green, C) DCX is shown in red, and D) the 3 channels were merged. Scale bar = 100 μm.

**Figure 5:**
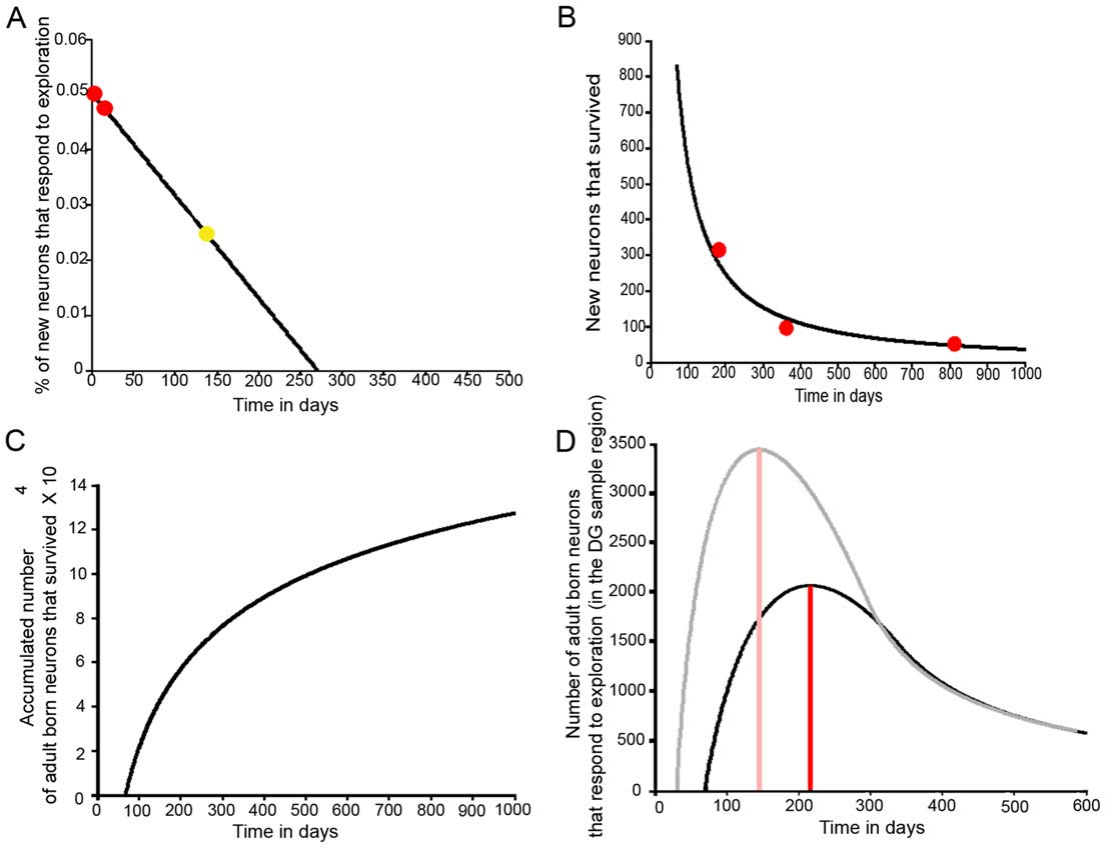
Contribution of adult-born neurons to spatial information processing: a hypothesis suggested by integrating our current data with that obtained previously. A) Linear regression plot calculated using the percentage of BrdU-positive cells that specifically respond to spatial exploration by expressing Arc from animals sacrificed on days 30 and 45 PBI (red dots), and those sacrificed 150 days PBI (yellow dot) [2]. Using this model we calculated the maximum length of time throughout the life span of the animal during which adult-born neurons respond to spatial exploration (time in days means age of animals in X axis for all graphs). The result suggests that after 301 (271+30) days, the neurons born in the adult brain may no longer respond to spatial exploration. B) The data obtained from Kuhn [29] was scaled to fit our BrdU-positive cell proportions, and a power model was used to calculate the number of neurons that survived at the different times through the animal's life. C) Cumulative number of new neurons that survived after 30 days including all the neurons born since 70 days post natal, when the animal is already mature. D) Total number of neurons born after DG development that contributes to the DG spatial exploration response over the course of the animal's life span. The red line shows the time (day 217) when the maximum number of newly incorporated neurons participates in the DG network response to spatial information processing. At this time, 2057 new neurons respond to spatial exploration, which represents 60% of the total DG granular cell population that responds to spatial exploration. The super-imposed graph (shown in light colors) represents the same calculation including the number of neurons born since postnatal day 1 that survive for 30 days. Notice in particular that the contribution to spatial information processing of neurons born after the DG development is complete increases through the early age of the animals, and after reaching its maximum, it rapidly decreases to a plateau reached in late adulthood.

Animals were administered BrdU on day 0 and sacrificed on day 1, 7, 15, 30, or 45, in each case thirty minutes after a 5-minute exploration session or cage control conditions (Fig. 1). Their brains were processed for triple immunohistochemistry for NeuN, Arc, and BrdU (also for DCX&BrdU and Arc&DCX), and semi-confocal and confocal microscopy images were taken for later analysis (see Fig. 2A&B, Fig 3, Fig. 4A, and Fig. 5D–H ).

We found that the expression of Arc occurs without stimulation in 1- to 15-day-old new granular neurons. A specific response to exploration was observed in ∼5% of the 30- to 45-day-old new-neurons. This temporal pattern of Arc expression in response to spatial exploration reflects the course of selective survival and integration in a network that processes spatial information. Ninety percent of the BrdU-positive cells expressing Arc in response to spatial exploration were located in DG layer 1 (L1), and no Arc expression was detected in new cells located in the subgranular zone (SGZ). Based on calculations using the current data and that obtained previously [2], we hypothesize that new neurons may no longer respond to spatial exploration after they are 301 days old, and that in a 7-month-old animal the majority (60%) of the neurons that respond to exploration were born during adulthood.

## Results

### Survival and migration of new neurons

The proportion of BrdU-positive cells in the DG upper blade varied significantly across the different maturation times (*F*_4,30_ = 29.771, *p*<0.001) (Fig. 2C). One day post BrdU injection (PBI), 0.26% of the entire population of granular DG cells included in the study had incorporated BrdU, and seven days PBI, the percentage of BrdU-positive cells (0.35%) was significantly higher (the Bonferroni test yields a *p*<0.01). At 15 days PBI, the proportion of new granular cells was 0.23%, which was significantly lower than on day 7 (*p*<0.01) but not statistically different from day 1. Thirty days PBI, the percentage of BrdU-positive cells was only 0.17% and was significantly lower than that found on days 1, 7, and 15 PBI (*p*'s<0.01). Finally, 45 days PBI, the percentage of BrdU-positive cells was only 0.15%, which was significantly lower than on days 1, 7, and 15 (*p*'s<0.01) but was not different from day 30. The ANOVA of the absolute BrdU-positive cell counts revealed a similar pattern through time (*F*_4,30_ = 89.772, *p*<0.001, Bonferroni test yields p's<0.01). These counts represent the average number of BrdU-positive cells found in each PBI group (Fig. 2D), without adjusting for the total population of granular cells included in the analysis.

In the animals sacrificed on day 1 PBI (Fig. 2E), 60% of the BrdU-positive cells were located in the sub granular zone (SGZ), 32% in DG layer 1 (L1), 7% in layer 2 (L2), and only 1% were found in layer 3 (L3). On day 7 PBI, 32% of the BrdU-positive cells were located in the SGZ, 54% in L1, 11% in L2, and 3% in L3. On day 15 PBI, 16% of the BrdU-positive cells were located in the SGZ, 58% in L1, 19% in L2, and 6% in L3. At 30 days PBI, 13% of the BrdU-positive cells were located in the SGZ, 54% in L1, 24% in L2, and 10% in L3. Finally, at 45 days PBI, 12% of the BrdU-positive cells were located in the SGZ, 51% in L1, 23% in L2, and 13% in L3.

The proportion of cells found in each DG layer was compared between the different PBI groups using a one-way ANOVA (Fig. 2E). The proportion of cells in the SGZ differed among groups (*F*_4,30_ = 176.086, *p*<0.001), where the proportion of BrdU-positive cells found in animals sacrificed on day 1 PBI was significantly different (using the Bonferroni post hoc correction) from all other groups (*p*<0.01). For L1, the proportion of BrdU-positive neurons differed significantly among groups (*F*_4,30_ = 47.687, *p*<0.001), and the post hoc analysis showed differences between animals sacrificed at day 1 PBI and all other groups (*p*<0.01). Differences were also found between animals sacrificed at day 15 and day 45 PBI (*p*<0.01). In L2, significant differences were found in the proportion of BrdU-positive cells among groups (*F*_4,30_ = 40.070, *p*<0.001), and the post hoc analysis revealed differences between animals sacrificed at day 1 PBI and the animals sacrificed 15, 30, and 45 days PBI (*p*<0.01); differences were also found between animals sacrificed at day 7 PBI and those sacrificed at days 15, 30, and 45 PBI (*p*<0.01); in L2, significant differences were found between animals sacrificed on day 15 and those sacrificed on day 30 PBI (*p*<0.01). In L3, significant differences were found among groups (*F*_4,30_ = 35.403, *p*<0.001), and the post hoc analysis revealed that most of the PBI groups differed from each other (*p*<0.01), except those sacrificed on day 1 vs. day 7 PBI and animals sacrificed on day 7 vs. 15 PBI.

The differences in the proportion of BrdU-positive cells located in the different regions between the animals sacrificed at different PBI times indicate that the newly incorporated cells migrate through the different DG layers.

### Sparse Arc expression in DG granular cells after spatial exploration

After spatial exploration (SE), the expression of the immediate early gene Arc was observed in ∼1.5% of the granular cell population, while in the cage control (CC) animals only ∼0.3% of the granular cells were classified as Arc positive. The percentage of cells expressing Arc was significantly different (*F*_1,33_ = 12.042, *p*<0.001) between CC and SE animals, demonstrating that a small population of granular neurons in the DG expressed Arc in response to a novel spatial exploration in an open box (Fig. 3A), as previously reported [22], [26], [27], and suggesting a sparse DG code for spatial information.

### Specific Arc expression response to spatial exploration develops in new neurons over a 30-day period

By using double staining, we observed that in animals sacrificed at early PBI times (1–15), BrdU is found mostly in DCX-positive cells and in late PBI time (30–45) primarily in NeuN-positive neurons (Fig. 3D–H). We also observed that Arc can be expressed in DCX positive cells. At early times (Fig. 4.) For these reasons we included all BrdU-positive cells found in the different DG layers in further analysis.

Arc expression in the BrdU-positive population differed across the different treatment groups (Fig. 3B). A 2-way ANOVA showed significant differences between days PBI (*F*_4,25_ = 3.79, *p*<0.02); between exploration and cage control conditions (*F*_4,25_ = 13.603, *p*<0.01), and among days and the behavioral treatment (*F*_4,25_ = 5.437, *p*<0.0027). One day PBI, Arc expression was observed in 1.3% of the BrdU-positive cells found in cage control animals (CC) and in 1.01% of the BrdU-positive cells found in animals exposed to spatial exploration (SE); these values were not significantly different. Arc was expressed in 2.1% of the 7-day-old new neurons from CC animals and in a similar percentage (1.56%) of the corresponding new neurons from SE animals. Moreover, the proportion of 7-day-old, BrdU-positive cells expressing Arc (BrdU+/Arc+ cells) did not differ from the proportion of new cells expressing Arc on day 1 PBI from either the SE or CC group. Fifteen days PBI, Arc was expressed in a similar proportion of new cells from CC animals (∼2.3%) and from the SE group (2.9%). Although Arc expression in BrdU-positive cells tended to increase from day 1 to day 15 PBI, this increase was not statistically significant. Importantly, these results suggest that Arc expression at this early time after these new neurons were born may not be driven by spatial behavior stimulation. In contrast, when new neurons were 30 days old, the proportion of BrdU+/Arc+ cells was significantly greater (*p*<0.01) in SE (∼5%) than in CC animals (∼1.5%). The proportion of BrdU+/Arc+ cells at 45 days PBI also differed significantly (p<0.001) between SE (4.8%) and CC animals (0.7%).

A repeated measures ANOVA was done on the percentage of cells expressing Arc at the different PBI times and revealed significant changes with time (*F*_1,5_ = 26.839, *p*<0.01), differences between SE and CC groups (*F*_4,4_ = 3.385, *p*<0.05), and also a significant interaction (*F*_4,6_ = 5.887, *p*<0.01), indicating that a specific Arc expression response to spatial exploration developed between 30 and 45 days after new neurons were born.

It is important to note that the proportion of BrdU+/Arc+ cells in response to exploration at 45 days PBI was significantly greater than the percentage of total granular cells expressing Arc in response to exploration (T _6_ = 7.995 *p*<0.001).

### Exploration-induced Arc expression in BrdU-positive cells is observed mainly in DG L1

The position of the BrdU-positive cells expressing Arc within the different granular layers of the dentate gyrus [8] was identified, and for each layer we calculated the proportion of BrdU-positive cells expressing Arc relative to the total population of BrdU-positive cells that express Arc. These proportions were compared between CC and SE groups (Fig. 3C) at all 5 time points when the animals were sacrificed. Significant differences were found between CC and SE animals in L1 (*F*_1,33_ = 8.754, *p*<0.01), where 89.3% of the BrdU-positive cells expressing Arc from the SE group but only 63.78% from the CC group were located. In L2, the proportion of Arc-expressing cells also differed significantly between CC and SE animals (*F*_1,33_ = 14.509, *p*<0.001) 29.56% of the BrdU-positive cells expressing Arc from the CC group were located in L2 as compared to 1.67% from the SE animals. In L3, no significant differences were found between groups. The MANOVA analysis revealed significant differences between the various layers in the proportion of BrdU-positive cells expressing Arc (Wilks lambda *F*_2,31_ = 7.183, *p*<0.01), and it is clear that the L1 region had the highest Arc expression in BrdU-positive cells. Importantly, BrdU-positive cells expressing Arc were not found in the SGZ.Using a Student's t-test we compared the proportion of BrdU+/Arc+ cells with the proportion of BrdU-positive cells among the different DG layers. Since the specific Arc expression response to exploration develops after 30 days, in this analysis we included only the animals sacrificed 30 and 45 days after the BrdU injection. The proportion of BrdU+/Arc+ cells were significantly higher than the proportion of BrdU-positive cells in L1 only for SE animals and not for CC animals (p<0.01). In contrast, in layer 2 of SE but not CC animals, the proportion of BrdU+/Arc+ cells was significantly lower (p<0.01) than the proportion of all BrdU-positive cells. No differences were found in layer 3.

### A hypothesis suggesting that adult-born neurons modify their contribution to spatial information processing throughout their life and the animal's life span

Here, we found that the proportion of new granular cells that responded to exploration 30 days after these neurons were born was 5%, and at day 45 PBI it was 4.8%. Previously, we reported that ∼2.8% of the 5-month-old, newly incorporated neurons responded to exploration [2]. This suggests that the likelihood of a new neuron to respond to behavioral exploration decreases with time [28]. In this study, we included ∼80,000 upper blade DG granular cells per animal, obtained from 450 µm in the antero-posterior axis. The whole sample region measured 1300 µm, and we calculated that it contained 231,000 DG granular neurons. From this population of neurons, 1.5% (3,465) responded to spatial exploration, a result similar to what has been reported previously [22], [26], [27].

We hypothesize that the probability of the adult-born granular neurons to respond to spatial exploration changes linearly with time:
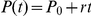
where P_0_ indicates the percentage of neurons that respond when they are 30 days old. We used a linear regression to estimate the parameters of our model (P_0_ = .054, r = −0.0002, *R^2^* = 0.9989, *p* = 0.0208), and the result is shown in Figure 5A, were the red circles represent our current data, and the yellow circle represents our earlier result [2]. The line represents the model, with which we calculate the probability that granular cells will respond to spatial exploration after they are more than 150 days old. Obtaining the intercept of the line to reach a probability of 0 by the following formula:
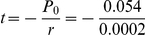



This suggests that 301 days after the new neurons were born, they no longer respond to spatial exploration. We also used the individual data from each animal to calculate this linear regression and obtained a significant regression (*P*_0_ = 0.0505, r = −0.0002, *R^2^* = 0.5101, *p* = 0.0135), validating the conclusion obtained with the average proportions. We acknowledge that a linear model with only 3 data points presents clear limitations, and further research is needed to add more data points between 45 and 360 days. This will allow us to determine if a linear model is adequate and will test the prediction that cells no longer respond to exploration after they are ∼1 year old.

The number of new granular neurons found 45 days after birth in the sampled regions was 345. Adult-born neurons that survived for 4 weeks remained stable for at least 11 months [8]; therefore, we can assume that the number of newly incorporated neurons detected at days 30–45 PBI represents the number of cells born on day 1 that will survive for the rest of the animal's life. However, the rate of cell proliferation varies across the animal's life span [29], and this modifies the number of stable new neurons throughout the life time.
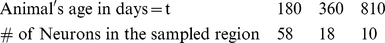
By using these data [29] we propose a power model:
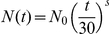



Since the DG is fully assembled after the first post-natal weeks [30], sexual maturity is reach after 6 weeks [31], and the neurons that survive for 4 weeks remain, we perform further calculations using the number of granular cells that were born and survived since post-natal day 70. N_0_ is then the number of new granular neurons that survive for 30 days. In order to estimate the parameters of our model, we calculate a linear regression. We choose ‘s’ such that the equation predicts the number of BrdU-positive cells that we detect after 45 days in the current work (Figure 2C&D). (*N(150) = 345*). The result, *N*_0_ *= 2218.2*, is shown in Figure 5B, where the red dots are the data and the solid line is the model. From these data we calculate the cumulative number of neurons that were born after the animal was sexually mature and that survived throughout the animal's life span, assuming that there is no mortality after 30 days of maturation [8].
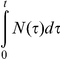



The cumulative number of adult-born granular neurons over the course of the animal's life span is shown in Figure 5C. In order to estimate the number of adult-born granular neurons that contribute to the DG response to spatial exploration throughout the animal's life span, we considered the number of neurons that are added through time (N(t)), and the probability that they will respond at different time intervals (P(t)) after they were added. In order to combine these measurements we used a convolution of the variables N and P.
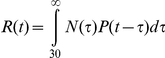



**Figure 6:**
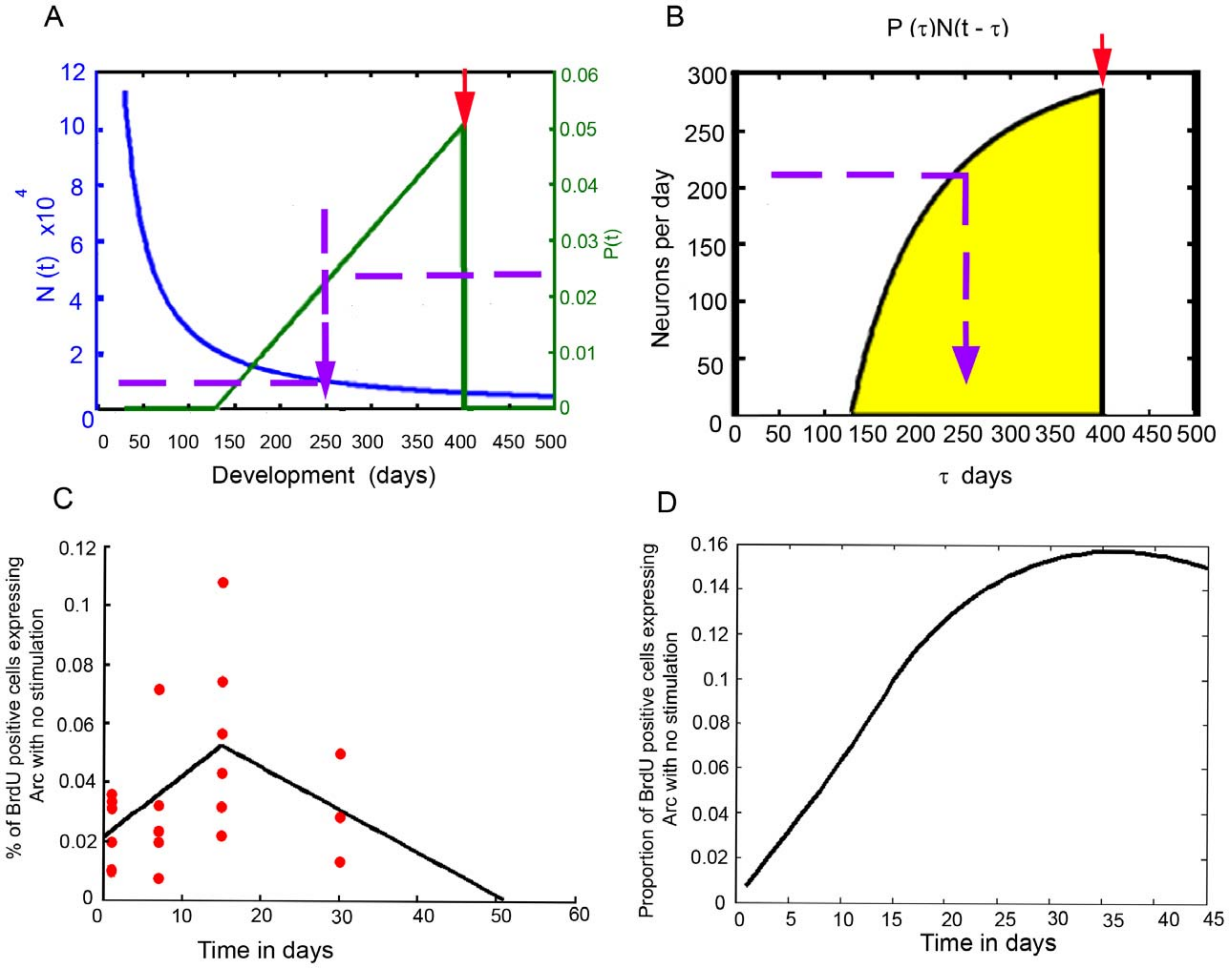
Illustration of the calculation procedure. A) As an example of our calculation that lead to the results found in Figure 6D, we can estimate the number of cells that contribute to the spatial exploration response at day 400 in the life of (red arrow), from cells born on day 250. We found ∼11,000 new neurons that were born on day 250 and survive for at least 30 days; when we multiplied this by the proportion of neurons that responds to exploration at day 400 (2%), we calculated that ∼220 cells respond to exploration (purple arrow in B); B) When all the new neurons are considered (area under the line in yellow) we have a total number of neurons that respond at day 400, from the cumulative number of new neurons at that time. C) To determine the background due to Arc expression during early development of new neurons, we calculated the probability of neurons to express Arc from 1 to 30 days, using 2 linear models. D) A similar convolution as the one used for our previous calculation was done to estimate the number of adult-born neurons found from 1 to 30 days PBI (Figure 5 C). Note that the maximum proportion of neurons that express Arc with no stimulation is ∼0.16%; for comparison, cage control animals present 0.31% of cells expressing Arc with no previous stimulation (Figure 5 A), and this represents ∼10% of the granular cells expressing Arc after spatial exploration.

This is the overlap level of N and the function of P transferred and inverted, which implies the number of new neurons 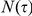 at time t (throughout the animal's life span) under the proportion *P* (*t*−τ) that contribute to spatial information precesing (see Fig. 6A and 6B for a graphic explanation). The resulting calculation showed that the time t, when the maximum number of neurons, that were born after the DG network was fully formed, contribute to spatial information processing, is at day 217, where 2057 of the adult-born neurons in the sample region are predicted to respond to spatial exploration (Fig. 5D). This represents 60% of the total granular cell population that responds to this behavior. If we include in our calculation all the neurons born after the animal's birth that survive for 30 days, the result suggest that at day 129 of the animal's life, 99% of the neurons that respond to exploration are neurons that were born post-natally (Figure 5D, shaded line).

We emphasize that this model is presented only as a new hypothesis suggesting that the neurons born in the adult mammal hippocampus change their contribution throughout both, their own and the animal's life span. These results, though inconclusive, should stimulate further studies that evaluate the relevance and contribution of newly incorporated granular neurons through time.

## Discussion

During the first 6 weeks, the average proportion of BrdU-positive cells found in the DG from all animals fell from ∼0.35% on day 7 to ∼0.15% on day 45, suggesting that ∼60% of the neurons detected on day 7 died over the course of the next 5 weeks.

It has been reported that in mouse, new neurons that survive more than 4 weeks represent 20% to 45% of the proliferating cells [2], [32] and that in rats, they represent 42 to 60% of the newly born neurons [32], [11]. In the present work, the number of BrdU-positive cells found on day 1 PBI was lower than the number of cells found on day 7 PBI. This may be explained by the fact that on day 1, only 12 hrs had passed since the last BrdU administration, and more new cells may have incorporated BrdU in the following hours [33]. This is consistent with an earlier report that used a single exposure to [^3^H]-methyl-thymidine and found more labeled cells on day 7 than on day 1 [34]. Here, we found that the proportion of granular cells born on one day that survive for 45 days is 0.15%, and that these cells may survive for 3 to 11 months [8], [32]; the result was similar using the absolute BrdU-positive cells counts. We calculated that the whole DG contains ∼1,200,000 granular cells, which is consistent with previous reports [35], [36] and suggests that the number of cells born in the DG during one day that survive is ∼1,800. A similar number can be calculated by using the estimated number of cells born each day in the adult rat DG [33] and subtracting the dying neurons [8], [32].

The location of the BrdU-positive cells among the different DG layers throughout time indicated that new cells migrate from the SGZ through the rest of the layers, as previously reported [8], [14]. The highest migration from the SGZ to DG L1 and L2 was reported to occur between day 7 and 14 [14]. We observed that from day 1 to 15 PBI, when expression of DCX is highest [12], the location of new cells changed significantly, primarily from the SGZ to DG L1 and L2, indicating that this new neurons migrate through the DG layers. Our results also show that a small proportion of BrdU-positive cells (∼10%) stay in the SGZ. Most (51%) of the BrdU-positive cells that survive for 45 days stay in DG L1, consistent with previous observations that ∼60% of the newly incorporated neurons were located in this layer [8]; at day 45 PBI the percentage of new cells that we found in L2 was 22.6%, and in L3 it was 13.5%, also consistent with the earlier report.

The proportion of DG neurons expressing Arc after exploration was 1.5%, significantly higher than that observed in the cage control animals and in agreement with the notion of a sparse code for spatial information processing in the DG [22], [26], [27].

The likelihood to observe Arc expression in BrdU-positive cells increases throughout new neuron maturation, as recently reported for other IEGs such as zif268 and cFos [32]. The highest IEG (zif268 and cFos) expression, after kainite-induced seizures, was observed at 4 weeks and remained stable at 10 weeks. The expression of zif268 induced by water maze training was maximal at 3 weeks; however, it is not clear if such expression was specifically induced by the behavioral treatment, since no behavioral controls were shown [32]. In our case, from day 1 until day 15 PBI the proportion of BrdU-positive cells expressing Arc was similar in cage control and exploration animals. Likewise, in animals that received LTP-inducing stimulation, Arc expression was reported in both the stimulated and the non-stimulated hemispheres in 1-day-old new neurons [25]. These data suggest that Arc expression in new neurons at an early stage of their development occurs independently of sensory or behavioral stimulation. We cannot suggest that this seemingly spontaneous or constitutive Arc expression [25] is independent of neuronal activity, but it may occur without synaptic stimulation since immature granular neurons do not respond to synaptic input or are “silent” at this early stage [14]. It is possible then, that these new neurons may be activated in response to paracrine BDNF release or other signals [37], which is an interesting idea since this time is critical for the maturation and synaptic integration of adult-born neurons. Arc expression at this early stage may be indicative of an ongoing biological process involved in the synaptic integration of adult-born neurons [25]. This hypothesis is supported by recent evidence showing that Arc increases the density of immature spines and regulates spine morphology [38]. Studying the role of Arc expression in young, adult-born granular neurons may help to understand the cellular mechanisms underlying the synaptic integration of these neurons.

On the other hand, Arc expression at these early stages may represent background noise in the system. For this reason, we calculated the number of new neurons that contribute to Arc expression in the general population. The result indicated that in cage control animals ∼50% of all the granular neurons expressing Arc may be new granular neurons born between 1 and 30 days before sacrifice (See Fig. 6C & D). However, this represents only ∼10% of the neurons expressing Arc in response to spatial exploration, suggesting that the background noise produced by this Arc expression in young new neurons contributes relatively little to the whole DG network response to exploration.

**Figure 7:**
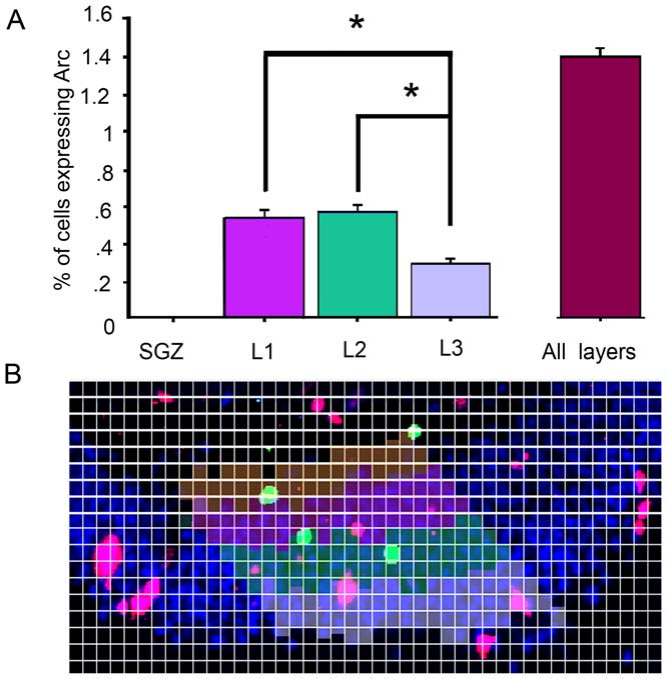
Dentate gyrus layer segmentation and Arc expression in the different layers. A) Shows a representative DG layer image segmented into 4 equidistant layers. NeuN-positive granular cells are shown in blue, Arc-expressing cells in pink, and BrdU-positive cells in green. The grid was used as a guide for the segmentation procedure, and the cells that were mainly (>50%) located in grids from a particular layer were considered to belong to that layer. The segmentation is represented by an overlay of translucent colors. In brown is the SGZ, in purple L1, in green L2, and in gray L3. B) Percentage of granular cells (from the general population) expressing Arc in the different DG layers after spatial exploration. The red bar is the proportion of cells expressing Arc in all layers.

At days 30 and 45 PBI, Arc expression in the BrdU-positive cells was significantly higher in animals allowed to explore than in cage controls, indicating that a specific response to exploration develops with maturation. Previously, it was reported that the unspecific expression of Arc in response to LTP stimulation lasted for about 28 days, but the number of BrdU-positive cells expressing Arc under these conditions increased on day 28 in the granular cell layer, and from day 14 onwards in the SGZ [25]. In contrast, we found no Arc expression in the BrdU-positive neurons located in the SGZ (Fig. 3C), indicating that new granular cells found in this DG region are not responsive to behavioral exploration and may not be functionally integrated into the behaviorally relevant network. This agrees with our own analysis of Arc expression in the different DG layers (Fig. 7A), where we did not find Arc-expressing cells after spatial exploration in the SGZ. This may suggest that migration from the SGZ to the DG granular layer is mandatory for new neurons to be able to respond to behavioral exploration and express Arc. The discrepancies with Kuiper's work may also be explained by the different stimulation methods and the animals that were used. It is possible, even in the non-stimulated hemisphere and in the SGZ [24], that a strong electrophysiological stimulus can activate the CA3c commissural projections [39], thereby inducing Arc expression in new neurons due to their enhanced plasticity [40]. Another possibility is that the functional integration of adult-born neurons into the hippocampal network may take longer in Sprague Dawley than in Wistar rats.

The enhanced plasticity of newly incorporated neurons (4 to 6 weeks of age) was confirmed in the present study with the observation that ∼5% of the new neurons found at 30–45 days PBI responded to exploration by expressing Arc, whereas only 1.5% of the total DG neurons responded. This difference is similar to a previous report [19] and agrees with our earlier findings [2], where a significantly higher proportion of 5-month-old new neurons than of total DG neurons expressed Arc in response to spatial exploration.

The timing for the development of this Arc expression in response to spatial exploration coincides with the dynamic cellular process that takes place in these cells [9], [14], [10] and reflects the course of selective survival and integration into a network that processes spatial information. Around the time new neurons become responsive [13], [14] and their glutamatergic and GABAergic input matures [13], [14], [15], [16], the expression of Arc rises and becomes specific. This suggests that the enhanced survival effect of behavioral stimulation soon after new neurons are born [41], [42] depends on a non-specific activation of DG neurons, probably related to BDNF expression [43], [44].

In animals exposed to spatial exploration, ∼90% of their BrdU-positive cells that express Arc were located in DG L1 compared to 60% in the cage control group. The distribution pattern of BrdU-positive neurons among the DG layers (Figure 2D) is similar to that observed for BrdU-positive neurons expressing Arc in cage control animals but notably different from the distribution of new neurons that respond to spatial exploration by expressing Arc. The observations that mature (28-day-old), new neurons found in DG L1 exhibit a well-defined dendritic structure, a conspicuous axonal projection extending towards the hilus, and spiny dendrites reaching the outer molecular layer [14] suggest that neurons located in L1 mature earlier, developing a functional response to the input, while neurons located in L2 and L3 develop these features later. This may explain why most of the adult-born neurons that are functionally integrated into the behaviorally relevant network are located in this inner layer, and suggests that the time required for migration may delay the functional integration of new neurons; thus, it is expected that neurons located in L2 and L3 will acquire the ability to respond specifically to spatial exploration at a time point later than 45 days, since Arc-expressing cells are found in all 3 granular layers with a bias towards those located in the inner DG (see Fig. 4B).

When we estimated the proportion of BrdU-positive cells that respond to spatial exploration by expressing Arc over the course of the animal's life span, we found a highly significant linear regression; thus, our hypothesis is that neurons born in the adult DG after day 301 do not respond to exploration anymore; this does not suggest that new neurons lose their integration within the network but rather that they can no longer be recruited into circuits that process spatial information (Fig. 5A), which may be related to a network mechanism that ensures that the DG code for spatial information can change. It is important to acknowledge that a non-linear model may also fit the data, and that more data points are required to demonstrate that our hypothesis is correct; however, the hypothesis is supported by the highly significant linear regression analysis and by the fact that the number of new neural units under the curve throughout the animal's life (Fig. 6D) agrees with the total number of Arc-expressing cells after exploration.

The hypothesis that old granular cells no longer process spatial information after a certain time agrees with the granular cell retirement hypothesis [45], which suggests that granular neurons process spatial information for a limited period of time. This satisfies an important requirement of the associative memory model from Treves and Rolls [46], which proposes that: “…during retrieval…, the mossy fiber input should be absent or strongly reduced…, in order not to blur the signal relayed by the perforant path”. Our data suggest that, instead of shutting down or reducing the DG input to the CA3 during retrieval, a few granular neurons retire every day, gradually changing the DG code of a particular experience. This hypothesis is of great interest, but its validity must be tested by further research with more time points, especially at late maturation times for adult-born neurons.

The model also suggests that in a 7-month-old animal, 60% ([5-month-old and 99%] if we include all neurons added postnatally) of the total granular neurons that responded to exploration should be neurons born after the DG was fully formed. This suggests that adult-born neurons may contribute more to spatial information processing than pre-natally born granular neurons. This idea was recently tested in mice that received the administration of the thymidine analog CidU either on embryonic day 18 or postnatal day 7 and another thymidine analog IdU on postnatal day 60 [47]. The results showed no differences in the proportion of granular cells responding to spatial behavior by expressing cFos. However, if we compare the time of analysis (Postnatal day 60) with our model (Fig. 5D) and consider that rats and mice present slight differences in the speed of maturation and life span [31], [48], it is possible that differences may be observed at later time points, congruently, other groups had recently found a higher proportion of adult born granular neurons responding to behavior compare to prenatally born granular neurons (Nora Abrous, Personal Communication). In any case, the model suggests that adult neurogenesis plays a pivotal role in spatial information processing, and that this contribution changes with time, which may explain cognitive changes related to hippocampal function over the course of the animal's life.

## Methods

### Subjects

Thirty-five adult male Wistar rats (4 months of age) were provided by the bioterium of our Institute. Rats were individually housed, had access to water and food *ad libitum*, and were maintained on an inverted 12 h∶12 h light-dark cycle, with lights on at 9:00 am. The animals were allowed to habituate to the room conditions and handled daily for at least 10 days before experiments began. The “bioethics committee” from our institute headed by Dr. Magdalena Giordano Noyola approved all the protocols and experimental procedures performed with the animals in the present study, which were done in accordance with international ethical guidelines for animal care and handling (ID:INEU/SA/CB/034).

### BrdU administration

Given that our goal was to accurately establish the date on which new neurons were able to respond to behavioral stimulation, we compared different 5-Bromo-2′-deoxyuridine (BrdU) (Sigma, St Louis MO ) administration procedures to maximize the number of newborn neurons detected on a single day. A dose of 200 mg/Kg, divided into four, 50-mg/Kg injections (diluted in 0.15 M NaCl solution) and administered every 4 hrs gave the highest number of BrdU-positive cells in the hippocampal dentate gyrus, as compared to other procedures tested. The first BrdU injection was at 9:30 am and the last was at 9:30 pm on the same day. After the BrdU administration, the animals remained undisturbed in their home cages until they were sacrificed after or without a 5-min spatial exploration session (see Fig. 1).

### Spatial exploration

In order to examine the response of new neurons to spatial exploration at different maturation times, 1, 7, 15, 30, or 45 days post BrdU injections (PBI), the animals were exposed to a 5-min exploration session (n = 5, 4, 4, 4, and 3 respectively) and were sacrificed 30 min later (Fig. 1). The exploration environment was an open square box, 70×70 cm, with 20-cm-high walls made of translucent acrylic. All the walls were covered with orange foamy paper, and the floor was partitioned into nine grids using black foamy paper strips. Each rat was fully covered with a white towel, then individually transported to the behavioral room, placed in the center of one of the grids in the apparatus, and moved to the center of a different grid every 15 s. This ensures that each of the grids was visited two or three times during the 5-min exploration session [22]. Immediately after exploration, the animal was placed back in its home cage and kept undisturbed. Cage control animals for each PBI group (n = 3) remained undisturbed in their home cages during the behavioral session and were sacrificed the same day and time as their respective exploration group.

### Brain extraction

Thirty min after the exploration session, each animal was killed by quick decapitation. The rat's brain was quickly and carefully extracted and frozen in 2-methylbutane (Sigma) by immersing it in a dry ice/ethanol slurry. The rat brains were stored at −70°C.

### Blocking and sectioning

Using a stainless steel matrix (Electron Microscopy Sciences, Hatfield, PA), brain hemisections including the whole hippocampi were obtained. From 8 to 10 brain sections were molded into a block with Tissue-Tec OCT compound® (Sakura Finetek, Torrance, CA), such that each block contained brains from all groups and the position of each group differed in each block. The blocks (4 total) were cryosectioned into 20-ìm-thick coronal sections at −18°C in a CM1850 Leica cryostat (Nussloch, Germany), captured on slides (Lauka, MEX) pre-treated with diethyl-polycarbonate (Sigma) solution, and kept in a sealed box at −70°C before the immunostaining procedure.

### Immunostaining

In order to maximize the detection of BrdU-positive cells expressing Arc, 30 to 40 serial sections from the dorsal hippocampus (Range between −2.60 to −4.3 from bregma) from each block were selected for the staining procedure. We used a triple immnunostaining protocol similar to that described previously to detect NeuN, Arc, and BrdU [2]. The tissue was fixed in 2% paraformaldehyde, pH 7.4, for 8 min at 4°C, washed in Tris-buffered saline (TBS), pH 7.0, and quenched in TBS with 2% H_2_O_2_ for 20 min. The sections were blocked for 40 min in tyramide signal amplification (TSA) kit blocking buffer (Perkin Elmer Life Sciences, Emeryville, CA). The tissue was then incubated sequentially with biotinylated mouse anti-NeuN antibody (1∶2000; Chemicon, Bedford, MA), with polyclonal rabbit anti-Arc antibody for the second detection (1∶500; a kind gift from Paul F. Worley's laboratory), and for the third detection with mouse anti-BrdU monoclonal antibody (1∶500 BD Biosciences México DF, México). After detecting NeuN and before Arc antibody incubation, the tissue was permeabilized with acetone/methanol (50∶50, v/v; Sigma) at 4°C for 15 min. For the detection of BrdU, the tissue was taken through a DNA denaturing procedure, consisting of an incubation with 50% formamide in 2×SSC buffer (Sigma) at 65°C for 2 h, washed in 2×SSC for 10 min, incubated in 2 N HCl at 37°C for 30 min, and washed in 0.1 M boric acid, pH 8.5, for 10 min. Biotinylated anti-NeuN was detected with the avidin-biotin A+B Vectastain amplification kit (Vector laboratories, Burlingame, CA) and the cyanine-5 (Cy5) TSA fluorescence system (PerkinElmer); before Arc detection, the A/B blocking kit (Vector laboratories) was used to block the remaining A+B, and the rabbit anti-Arc was detected with biotinylated anti-rabbit antibody (Vector laboratories), amplified with the A+B Vectastain amplification kit, and finally visualized with the Cy3 TSA fluorescence system (PerkinElmer). Mouse IgG from the first detection was blocked using the mouse-on-mouse blocking kit (Vector Laboratories) before detection of BrdU. The mouse anti-BrdU antibody was detected with a biotinylated anti-mouse antibody in which the signal was amplified using an A+B Vectastain amplification kit, and finally observed using the FITC TSA fluorescence system (PerkinElmer).

In order to determine the neuronal lineage of BrdU-positive cells detected early in their maturation, a double staining for doublecortin (DCX) and BrdU was done. In this case, BrdU detection as described above was carried out first, and DCX was detected afterwards using a goat anti-DCX antibody (1∶200, Santa Cruz®), then amplified with A+B Vectastain amplification kit, and revealed with Cy3. We also performed double immunohistochemistry for Arc and DCX combining the methods described above. No staining was observed in the absence of the primary or secondary antibodies, for all antigens.

### Imaging and Analysis

The MosaiX module for the APOTOME system (Carl Zeiss, México, DF. México) with the 25×/0.80NA LCI Plan-Apochromat oil immersion objective was used to obtain whole dentate gyrus (DG) mosaic image stacks (with 1.5-ìm optical Z sections). About 8 to 12 individual image stacks were collected and assembled by the MosaiX system (Carl Zeiss) for each DG. About twenty-three whole DG mosaics, taken from the serial stained sections, were imaged for each animal, corresponding to a dorsoventral length of ∼460 ìm from the dorsal hippocampus. Note that only those sections that were optimally stained were included in the analysis. The most anterior section was ∼6.0 mm and the most posterior was ∼4.7 mm from the interaural plane [49].

Using the Metamorph imaging software, a 2D image was constructed using the middle plane image from each DG MosaiX stack. This was used as the reference image, in which the DG granular layer was partitioned into 4 layers [8] (see Fig. 7B for a visual description of the segmentation); one represented the subgranular zone (SGZ), and the other 3 represented the inner (L1), middle (L2), and outer (L3) part of the DG granular layer. It is important to note that the DG granular layer thickness varies across the length of the blade; for this reason, the proportion was adjusted throughout the whole length of the DG granular layer, assuring that the inner, medial, and outer DG granular layers always represented 33% of the whole layer thickness in order to analyze the position of the new granular neurons within the DG granular layer. Meanwhile, the MosaiX stack was used to identify the NeuN-positive cells (image in blue), the cells that had incorporated BrdU (image in green), and those expressing Arc (image in red). The BrdU-positive cells co-localized with NeuN, particularly in the animals sacrificed 15 or more days after BrdU administration; in animals sacrificed earlier, BrdU co-localized primarily with DCX (Fig. 2A and B). Nearly all of the BrdU-positive cellsco-localized with DCX on days 1 to 15, and most of them co-localized with NeuN from day 30 onwards. These expression time points are similar to those reported earlier [12], in which the DCX or NeuN cells represented ∼90% of the total BrdU-positive population. Moreover, we found that in animals sacrificed at early time points (1–15 days PBI) during the maturation of these new neurons, Arc expression was found in DCX cells (Fig. 7A). For this reason, we included all BrdU-positive cells in the study, and the NeuN staining was used to delineate the DG granular layer. The Arc-positive cells were considered to be the activated neurons. Each cell was classified as BrdU-positive, Arc-positive or positive for both BrdU and Arc, and its classification was marked in the reference image, according to its position in the granular layer. It is important to clarify that the image stack was used to properly classify each cell as Arc-positive or BrdU-positive; additionally, when a cell was classified as both BrdU-positive and Arc-positive, a 40× confocal image stack was projected as a 3D image to confirm this classification (see Fig. 3D to H). The absolute BrdU-positive cell counts are the summation of all BrdU-positive cells found in all DG images from each animal (∼23 images per animal), and an average per group was obtained. After classification, the DG granular layer volume was calculated in the reference image, using a 40× confocal image in which the useful planes were obtained that, in combination with the area of the DG granular layer, were used to calculate the depth of the tissue (see Figure 2C and D). The total number of granular cells within the volume of the DG granular layer was estimated for each animal, as described before [25], [27]. We estimated that about ∼80,000 granular neurons per animal were included in the analysis. Using the total number of granular cells per animal, the proportions of BrdU-positive cells, Arc-expressing cells, and BrdU-positive Arc-expressing cells were calculated.

### Statistics

One-way ANOVA with Bonferroni as a Post-hoc test, MANOVA, or a student's t-test was used where appropriate to compare the proportion of BrdU-positive, Arc-expressing, and BrdU-positive Arc-expressing cells in the different conditions and throughout the DG granular layer.
